# Importance of Second-Look Surgery in a Newborn Presenting With Early Malrotation

**DOI:** 10.7759/cureus.26551

**Published:** 2022-07-04

**Authors:** Varun Kulkarni, Kiran Khedkar, Harshal Ramteke, Yashwant Lamture, Tushar Nagtode, Anamika Giri

**Affiliations:** 1 Department of General Surgery, Jawaharlal Nehru Medical College, Datta Meghe Institute of Medical Sciences, Wardha, IND; 2 Department of Pediatric Surgery, Jawaharlal Nehru Medical College, Datta Meghe Institute of Medical Sciences, Wardha, IND; 3 Department of Medicine, Jawaharlal Nehru Medical College, Datta Meghe Institute of Medical Sciences, Wardha, IND

**Keywords:** resection, impending perforations, midgut rotation, contrast study, volvulus

## Abstract

Intestinal malrotation refers to the abnormal positioning of the intestines due to a deviation from normal developmental stages. Volvulus is seen in 60%-70% of neonates diagnosed with intestinal malrotation. We are reporting a case of s six-day-old male who presented with multiple episodes of bilious vomiting and constipation and had malrotation of intestines with midgut volvulus. After a contrast upper GI study, the patient was taken for exploratory laparotomy, and extensive patches of an early stage of bowel ischemia were observed; resection was avoided at this stage. In the second-look surgery, all the gangrenous bowel loops were resected, and anastomosis was done.

## Introduction

Intestinal malrotation (IM) points towards all the deviations from the normal development of intestinal attachments and position [1]. Normal development of the midgut in the embryonic life results in rapid growth of midgut resulting in physiological umbilical herniation during the sixth week of gestation. During week 10 through 11, the gut starts having its 270° rotation, which is counterclockwise and around the axis of superior mesenteric artery and reenters the abdomen. Fixation occurs by 12^th^ week. This process is called as rotation of midgut. Any abnormality in this process leads to malrotation. One in 6000 live births show the incidence of the malrotation, which is symptomatic [2]. More than half of the patients present with the symptoms during the first month of life, and virtually all have bile-stained vomiting [3].

Evidently a case highlighting the benefits of the second look surgery in a case of malrotation has a scarcity of the literature about the association.

## Case presentation

**Figure 1:**
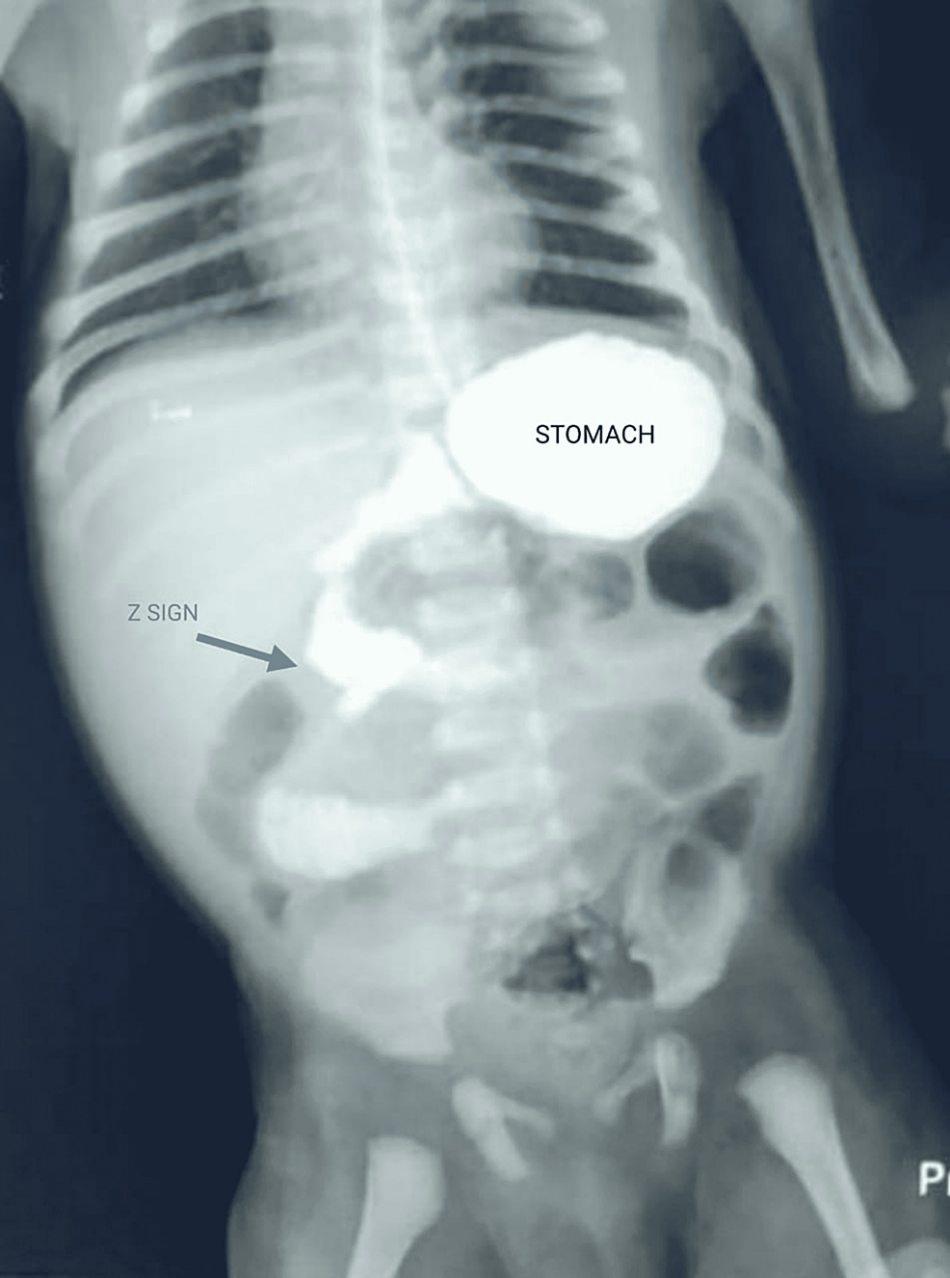
Upper gastrointestinal contrast study Arrow - Z sign due to duodenojejunal flexure on the right side of spine

A six-days-old, 3.3 kg child born to a primigravida mother at term gestation via normal vaginal delivery was brought to the emergency department. The child was well till the fifth day of life, when he presented with multiple episodes of bilious vomiting and constipation. He was vitally stable, pink, warm, but irritable on examination. His abdomen was distended, and the rectum was empty with normal anal canal mucosa. The initial management was gastric decompression with a nasogastric tube, administration of intravenous (IV) fluids and IV antibiotics. Abdominal plain radiograph and ultrasound were inconclusive, hence proceeded with the upper gastrointestinal contrast study in the neonatal intensive care unit. The findings were duodenojejunal (DJ) flexure was on the right side and Z sign with no dye passing into the distal intestine, confirming the diagnosis of malrotation with midgut volvulus. The decision was to do an emergency exploratory laparotomy (Figure 1).

**Figure 2:**
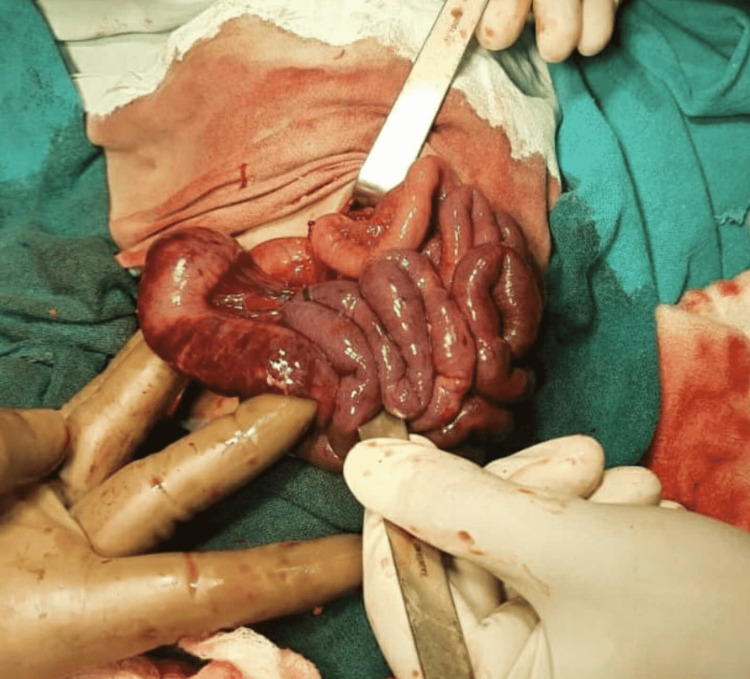
Bowel loops with doubtful vascularity

An exploratory laparotomy was performed for malrotation with midgut volvulus. Intraoperatively chylous fluid was seen, malrotation with midgut volvulus is seen and grossly ischemic small bowel loops were noticed. After derotation of the volvulus, the blood supply of proximal 4 cm of the jejunum and distal 3 cm of the ileum appeared normal, but the viability of the rest of the bowel segment was doubtful. Ladd's procedure was performed, but resection of the ischemic bowel was avoided at this point in time. Postoperatively the patient was observed closely for any signs of peritonitis or perforation. The patient was having episodes of dark brown colored aspirate through the nasogastric tube and had not passed stools (Figure 2).

**Figure 3:**
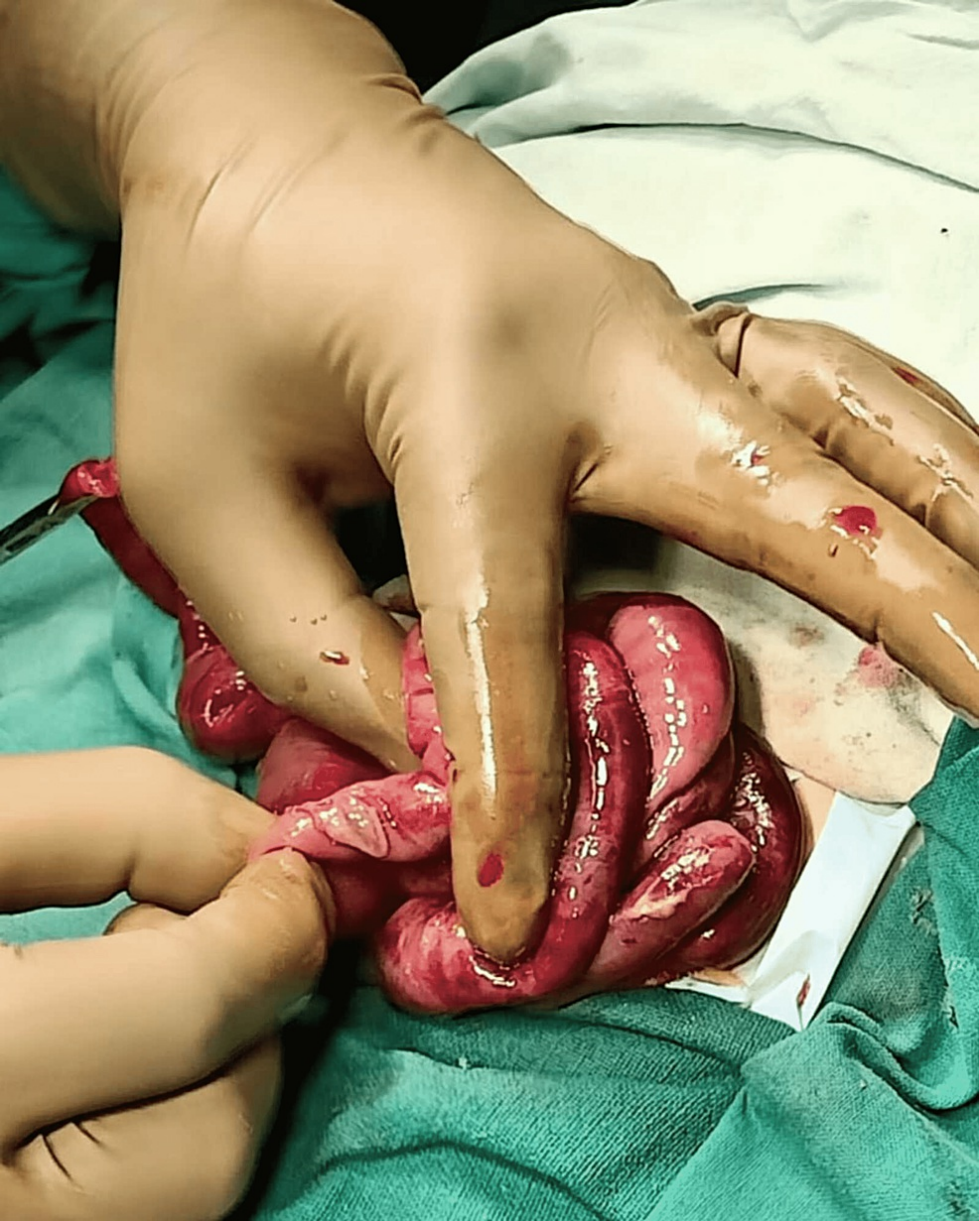
Second-look surgery, impending perforation

A second look surgery was performed 48 hours after the first operation, which attempted to correct the malrotation. There was no peritoneal contamination, but few impending perforations were seen. Most of the bowel loops were found to be gangrenous intraoperatively. The obvious gangrenous bowel loops were resected. The rest of the bowel was checked for vascularity. The area with no or poor blood flow was resected. Approximately 35 cm of the bowel from the duodenojejunal (DJ) junction to the ileocolic junction could be preserved (Figure 3).

**Figure 4:**
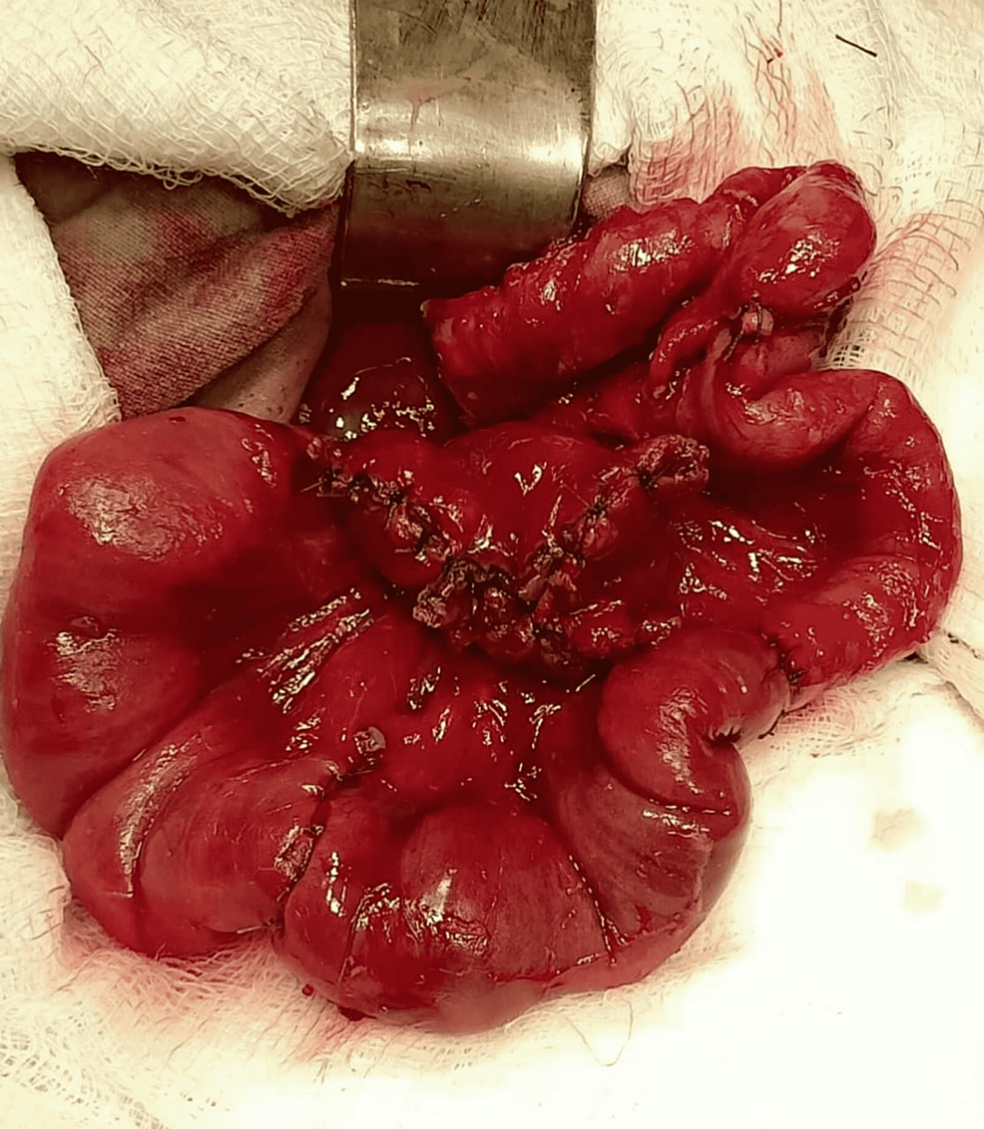
Anastamosis post resection

Postoperatively, the patient was kept nil by mouth (NBM) for five days and was kept under close observation for early detection of signs of peritonitis. The patient started passing stools after about five days and was breastfed on day six post-operatively. The patient was discharged once adequate feeding was established and was on close follow-up with growth monitoring (Figure 4).

## Discussion

The most dreaded complication of malrotation is volvulus. Volvulus is an entity where there is the rotation of the gut along the stalk of its mesentery, which is seen in 60%-70% of neonates diagnosed with intestinal malrotation. It can also present with strangulation in about 15% of cases [4,5]. The patient may land up with intestinal ischemia, intestinal necrosis, septicemia, and short bowel syndrome if there is any delay in the diagnosis of this condition [6]. The signs and symptoms in neonates are difficult to distinguish from those of duodenal stenosis. The duodenal stenosis presents with bilious vomiting and epigastric distension, which usually resolves after aspiration by nasogastric tube or episodes of vomiting. Pain or irritability cannot be labeled as a significant clinical feature in neonates. It can be considered significant in toddlers and older children. Bilious vomiting in a neonate raises the suspicion of intestinal malrotation with midgut volvulus until proven otherwise. Plain abdominal X-rays are usually normal but can show dilated duodenum with a fluid level and an ample amount of gas in the distal bowel loops [2,4], but in our case, gas was seen in the distal small bowel with mild dilatation. For any child presenting with bilious vomiting, the investigation of choice is upper gastrointestinal contrast study and should be done immediately [4]. But this study is rarely performed as most of the treating physicians are not familiar with the procedure. In our case, plain radiograph of the abdomen and ultrasonography were non-diagnostic, but the patient showed clinical features of midgut volvulus; hence, an upper GI contrast study was done, which confirmed the diagnosis, and emergency surgery was performed, thus avoiding further delay.

Second look surgery is usually administered in cases of necrotizing enterocolitis where there are numerous patches of the involved pathological bowel having doubtful outcomes [7]. This method was advised in the cases of malrotation in literature but is not as widely used. We, in our rural setup, have done this method of second-look surgery in the case of malrotation.

By this technique, approximately 30cm of the bowel loops could be preserved additionally by the second look surgery rather than going aggressively in the first sitting where most of the bowels would have been resected. Thus, the second look surgery was much more beneficial for the patient as the patient is now tolerating the oral feeds and is passing normal stools and flatus. The patient, in this case, has a high chance of failure to thrive, short bowel syndrome, and various micronutrient deficiencies. The patient will need lifestyle modifications. But the second look surgery approach provided the newborn with approximately 30cm of extra bowel, which can prove to be vital for the patient. 

## Conclusions

More studies should be performed in order to highlight the benefits of the second-look surgery in conserving more length of the bowel after an initial exploration. We aim to educate and encourage fellow surgeons to opt for this approach for better outcomes and to provide the patients a healthier quality of life. Upper GI contrast study in a neonatal bowel obstruction is simple to perform and read even if there is an unavailability of trained radiologists or high diagnostic accuracy. This can be performed even in a rural set up having limited resources. 
